# Integrated Surveillance for Human and Animal Brucellosis in Kenya: A Predictive Analysis

**DOI:** 10.3390/tropicalmed10120344

**Published:** 2025-12-09

**Authors:** Samuel Kahariri, Lian F. Thomas, Bernard Bett, Marianne W. Mureithi, Anita Makori, Brian Njuguna, Samuel Kadivane, Dennis N. Makau, Nyamai Mutono, S. M. Thumbi

**Affiliations:** 1 Directorate of Livestock Policy, Research and Regulations, Ministry of Agriculture and Livestock Development, Nairobi 00625, Kenya; 2 International Livestock Research Institute, Old Naivasha Road, P.O. Box 30709, Nairobi 00100, Kenya; lian.thomas@ed.ac.uk (L.F.T.); b.bett@cgiar.org (B.B.); 3 Centre for Epidemiological Modelling and Analysis, University of Nairobi, P.O. Box 19676, Nairobi 00202, Kenya; anitamakori99@gmail.com (A.M.); brian.njuguna@cema.africa (B.N.); mutono.nyamai@uonbi.ac.ke (N.M.); thumbi.mwangi@wsu.edu (S.M.T.); 4 Department of Medical Microbiology & Immunology, Faculty of Health Sciences, University of Nairobi, Nairobi 00100, Kenya; marianne@uonbi.ac.ke; 5 Royal (Dick) School of Veterinary Studies, University of Edinburgh, Easter Bush Campus, Midlothian EH25 9RG, UK; 6 Division of Disease Surveillance and Response, Ministry of Health, Nairobi 00100, Kenya; kadivane75@gmail.com; 7 Department of Biomedical and Diagnostic Sciences, College of Veterinary Medicine, University of Tennessee, Knoxville, TN 37996, USA; dmakau@utk.edu; 8 Department of Public Health, Pharmacology and Toxicology, Faculty of Veterinary Medicine, University of Nairobi, Nairobi 00100, Kenya; 9 Paul G. Allen School for Global Health, Washington State University, Pullman, WA 99164, USA; 10 Institute of Immunology and Infection Research, University of Edinburgh, Ashworth Laboratories, Kings Buildings, Edinburgh EH9 3FL, UK

**Keywords:** brucellosis, surveillance, zoonotic, time series, forecast

## Abstract

Brucellosis is a bacterial zoonotic disease which poses a significant public health challenge globally. In Kenya, it is a priority zoonosis, causing morbidity and losses in humans and animals. Here, we used monthly surveillance data from 2014 to 2022 from the official human and animal health surveillance databases. We conducted spatiotemporal analysis, tested associations between human and animal brucellosis using Time Series Linear Models, and forecasted the incidence of human brucellosis for twelve months using Seasonal Autoregressive Integrated Moving Average (SARIMA) models. Our analysis revealed a significant disparity in brucellosis cases, with a much higher cumulative number of human cases (4,688,787) compared to animal cases (1214). Human incidence depicted a relatively stable trend, with occasional fluctuations. However, cattle and camel incidences displayed sporadic peaks and troughs. Only cattle brucellosis was significantly associated (estimate: 0.355; 95% CI: 0.004 to 0.707) with human brucellosis. SARIMA models demonstrated reasonable predictive accuracy for human incidence, but incorporating animal data did not significantly improve model performance. Our study highlights the weaknesses in the existing surveillance systems and the need for comprehensive evaluation of the systems and implementation of integrated surveillance to address gaps in surveillance, improve the accuracy of predictive analysis, and enhance early detection for zoonotic diseases.

## 1. Introduction

Brucellosis is a globally widespread zoonotic disease with an estimated annual incidence of 1.6–2.1 million cases, the majority of which are in Africa and Asia [1]. In livestock, brucellosis causes large economic losses due to abortions, reduced fertility, and subsequent reduced milk production over an animal’s life span [2]. Transmission of brucellosis from animals to humans is primarily through contact with materials from infected animals and consumption of unpasteurized dairy products [3,4,5]. Brucellosis is mainly caused by different *Brucella* species with variable virulence. Worldwide, *Brucella melitensis* is the most common species causing human brucellosis, typically acquired from sheep and goats, while *B. abortus* is predominantly found in cattle [6]. In Kenya, brucellosis is listed as one of the top-five priority zoonotic diseases due to its high prevalence, public health importance, and impact on livestock production [7]. Kenya has developed a Brucellosis National Prevention and Control Strategy that focuses on strengthening integrated surveillance, enhancing coordination mechanisms, harmonizing One Health policy and institutional frameworks, creating awareness, administering targeted livestock vaccinations, standardizing human and animal brucellosis testing, and enforcing biosecurity and biosafety measures towards progressively controlling the disease in animals and humans by the year 2040 [8].

Several studies in Kenya have provided important epidemiological information about brucellosis. These studies have included estimates of incidence in humans in pastoralist settings [7], variations in the prevalence of human and animal brucellosis across the country [9,10,11,12], associations between animal and human brucellosis from linked household studies [9,10,13], and identifications of the circulating Brucella species in different livestock hosts [14,15]. The majority (85%) of research studies on zoonotic diseases in East Africa between 1920 and 2017 primarily focused on endemic diseases and were mainly conducted as cross-sectional studies. This highlights the need to expand the scope and improve the quality of these studies to fully understand and address the potential impacts of zoonoses [16]. There has been little focus on developing early warning systems for brucellosis using animal and human health data. We found no studies that have evaluated data from routine human and animal surveillance information systems to test the association between reported human and animal brucellosis and to determine whether animal cases can be used to forecast human cases in Kenya. This study also provides empirical evidence on the status of the existing human and animal health surveillance systems and provides important insights into potential initiatives for improving the early warning system for zoonotic diseases.

Analysis of surveillance data can provide insights for critical planning, detection of cases and case surges, and managing disease outbreaks effectively. An effective disease surveillance system is essential for enhancing rapid detection of disease outbreaks or health events before transmission within or across species that can cause loss of lives [17,18]. The key objective of surveillance is to provide data to guide interventions [19]. Establishment of a fully integrated One Health surveillance system will result in improved prevention, detection, and response to disease threats and prevent spillover of most infections to humans [18]. One Health surveillance should track multiple indicators of health challenges, combined with traditional disease-based surveillance, and must include surveillance of disease drivers to improve prevention and mitigation of spillover [18]. Scientific analysis and prediction of incidence of brucellosis cases in humans can benefit from animal and climatic data and assist in decision making for prevention and control measures [20].

In Kenya, public health facilities across the country are equipped with varying capacities for diagnostic tests, ranging from basic to complex, ensuring comprehensive diagnostic services at different healthcare levels. On the other hand, the animal health surveillance system primarily depends on only eight veterinary laboratories for disease confirmation [21]. These laboratories are sparsely distributed, and veterinary practitioners may incur expenses when submitting samples, which can discourage sample collection and testing.

In this manuscript, we determined the spatial–temporal patterns of reported cases of brucellosis in the national human and animal surveillance systems and the association between animal and human brucellosis cases, and performed short-term forecasts of human brucellosis across the country. These analyses improved our understanding of the burden of brucellosis in animals and humans over time across the country and of the utility of geographically linked animal and human national surveillance systems as an early warning system for zoonotic infections such as brucellosis. This work focuses on examining the current operational performance, capabilities, and limitations of the existing national surveillance systems to inform strategies for integrated One Health surveillance.

## 2. Materials and Methods

### 2.1. Data Sources

The analyses in this study used routinely collected data reported to Kenya’s national human and animal health surveillance systems between 2014 and 2022. These surveillance data reflect the real-world operation of Kenya’s reporting infrastructure. We obtained data on the human cases of brucellosis from the Kenya Health Information System (KHIS) as monthly aggregates of cases reported from health facilities [22]. These data are collected manually into registers by the records officers at the facility level and tallied into aggregates which are submitted on a weekly basis to the subcounty for entry into the KHIS.

Data on brucellosis cases in animals (cattle, sheep, goats, and camels) were obtained from the Kenya Animal Bio-surveillance System (KABS) [23] and the Directorate of Veterinary Services repositories, which compile data collected through all official platforms. All the counties are expected to report cases on a near real-time basis using the available official channels and tools. The reported cases of brucellosis are based on both clinical and laboratory diagnosis for all animal species under surveillance. The data collected from both human and animal health surveillance systems spanned from January 2014 to December 2022. Clinical diagnoses were made by qualified health and veterinary professionals in all 47 counties of Kenya, without the use of laboratory facilities. In contrast, laboratory diagnoses involved the use of laboratory tests or postmortem examinations to identify cases of brucellosis.

Additionally, we used the 2019 census data obtained from the Kenya National Bureau of Statistics (KNBS) to estimate human and animal populations [24] (34,935,548 cattle, 34,259,212 sheep, 55,480,306 goats, and 5,942,222 camels). The annual human population estimates for each county were projected using annual population growth rate estimates from KNBS [24]. Data cleaning was performed by the study team using R statistical software version 4.4.0. Any discrepancies, omissions, duplications, or outliers in the data were reviewed and resolved in consultation with relevant surveillance officials.

### 2.2. Data Analysis

#### 2.2.1. Estimating Incidence

We computed the monthly incidence rate of human brucellosis for each county using the number of cases reported during the month divided by the estimated human population in the county during the same month (assumed to be equivalent to the annual population estimate for the county). To estimate the brucellosis incidence for each of the selected animal species, we used the reported cases in the animal species during the month and the estimate of their respective populations based on the 2019 animal census data, since this provides the most credible and recent estimates. Brucellosis incidence for both humans and animals was expressed as cases per 1,000,000 population.

Temporal trends and spatial patterns of brucellosis incidence in humans and livestock species were visualized using time series graphs and choropleth maps.

#### 2.2.2. Test of Association Between Animal and Human Brucellosis Cases

We tested the associations between reported human cases of brucellosis and brucellosis cases for each animal species (cattle, camels, sheep, and goats), and the combined animal cases from all species at both national and county levels. To assess these associations, we fitted Time Series Linear Models (TSLMs) using lags ranging from 0 to 6 months, while controlling for trends and seasonality. The lags were set in consideration of the brucellosis incubation period, potential delays in reporting, and possible exposure dynamics. This approach aimed to investigate the association between human brucellosis incidence and animal brucellosis incidence.

We calculated the Root Mean Square Error (RMSE), Mean Absolute Error (MAE), and Mean Absolute Percentage Error (MAPE) for all the lag models to determine which model best fit the data. We confirmed this by checking residual plots and autocorrelation function plots to make the final selection of the lag.

#### 2.2.3. Forecasting of Human Brucellosis Cases

Let $Y_{t}=1,2,3,\ldots i$ denote the incidence of human brucellosis at time t. To model and predict $Y_{t}$, we analyzed its temporal patterns, which may have included trends, seasonality, and randomness, using time series methods. We first tested for stationarity using the Augmented Dickey–Fuller (ADF) unit root test, which indicated that $Y_{t}$ was non-stationary, suggesting a long-term trend. Stationarity, where the mean and variance of the series remain constant over time, is a prerequisite for many time series models.

A widely used method for modelling time series data is the Auto-Regressive Integrated Moving Average (ARIMA) model, denoted as ARIMA(p, d, q), where *p* represents the number of lagged observations in the autoregressive component, *d* is the degree of differencing required to achieve stationarity, and *q* is the order of the moving average component.

If $Y_{t}∼ARIMA(p,d,q)$, then $\Delta^{d}Y_{t}∼ARMA(p,\;q)$. The general equation for ARMA(p,q) is

$$\phi (B)\Delta^{d}Y_{t}=\theta (B)\epsilon_{t}$$

where $\phi (B)\;=\;(1-\phi_{1\;}B\;-\;\phi_{2}B^{2}-\;\cdots \;-\;\phi_{p}B^{p})$ is the autoregressive (AR) polynomial, $\theta \left(B\right)=\;\left(1+\;\theta_{1\;}B+\;\theta_{2}B^{2}+\cdots +\theta_{q}B^{q}\right)$ is the moving average (MA) polynomial, B is the backshift operator, $\Delta^{d}$ is the differencing operator, and $\epsilon_{t}$ is a white noise error term with zero mean and constant variance.

To address possible seasonal patterns in brucellosis incidence, we employed a Seasonal ARIMA (SARIMA) model, which extends ARIMA to capture both trends and seasonal fluctuations, making it suitable for high-accuracy, short-term predictions of diseases like brucellosis. The SARIMA model, denoted as $ARIMA(p,d,q)\times {\left(P,D,Q\right)}_{s}$, includes additional seasonal parameters P, D, Q, and s, where P is the order of the Seasonal AR component, D is the degree of seasonal differencing, Q the order of the seasonal moving average component, and s is the seasonal period. The SARIMA model is expressed as

$$\Phi (B^{s})\phi (B){\Delta_{s}}^{D\;}\Delta^{d}Y_{t\;}=\Theta (B^{s})\theta (B)\epsilon_{t}$$

where Φ(.) and Θ(.) are the polynomials of order P and Q, respectively.

The initial SARIMA model solely incorporated human incidence, excluding the covariate, whereas the subsequent SARIMA model integrated animal incidence as the exogenous variable. The stepwise method was employed to identify the optimal model, with selection guided by the Akaike Information Criterion (AIC). The model characterized by the lowest AIC value was selected as the most suitable model for forecasting the human incidence.

A three-month lag for combined animal incidence was used because it best fit the data and was therefore considered most suitable for predicting the human incidence as an exogenous variable.

**Figure 1:**
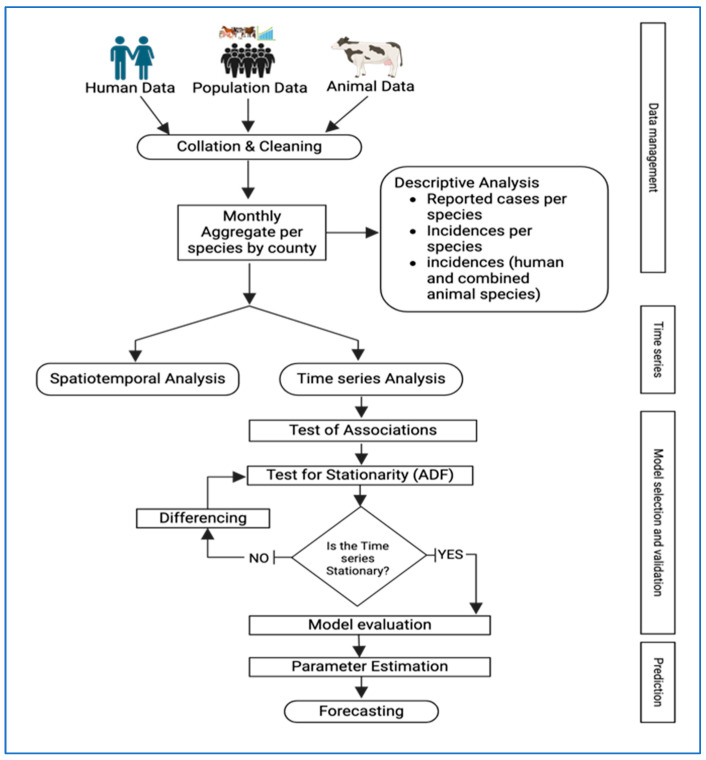
Summary of methodology and approach used to undertake the spatiotemporal analysis and forecast human brucellosis incidence for 2014–2022. (Created in BioRender. Manyu, R. (2025) https://BioRender.com/nbmw39m.).

To forecast the number of human brucellosis cases over a 12-month period, we utilized SARIMA models. We used data aggregated monthly at the national level. We divided our data into two sets: training data from 2014 to 2021 and testing data from January to December 2022 (with an 80% to 20% split). We calculated the Root Mean Square Error (RMSE), Mean Absolute Error (MAE), and Mean Absolute Percentage Error (MAPE) to determine which model performed best in forecasting human incidence in 2022. After testing the models, we retrained the best model using the complete dataset from 2014 to 2022 and then generated forecasts for 2023. A summary of the methods is presented in Figure 1 below. All analyses were conducted using the R statistical programming software version 4.4.0 [25].

## 3. Results

Over the entire period of 2014–2022, the number of reported human cases was significantly higher than the number of reported animal cases, with human cases totalling 4,688,787 compared to 1214 animal cases over the same period. Most of the animal cases were in goats, at 54.1% (656/1214), and the rest of the species-specific distribution was as follows: 35.2% cattle (427/1214), 8.2% sheep (99/1214), and 2.6% camels (32/1214). The estimated average incidence of human brucellosis varied widely between counties and ranged from 3 cases per 1000 people in Kilifi County to 399 cases per 1000 people in Elgeyo Marakwet County. Other counties with high incidence rates (per 1000) included West Pokot (375) and Nandi (304). In terms of the lower end, counties such as Kilifi (4), Lamu (7), and Mombasa (6) reported significantly lower incidences.

The incidence of cattle brucellosis showed notable regional differences, ranging from 0 cases per 1,000,000 cattle in several counties to 235 cases per 1,000,000 cattle in Mombasa County. Eight counties had zero reported cases of brucellosis in cattle. Camel brucellosis incidence was relatively rare, with higher rates in specific regions. The incidence ranged from 0 cases per 1,000,000 camels in 38 counties to 122 cases per 1,000,000 camels in Tana River County. Sheep brucellosis incidence was generally low, ranging from 0 cases per 1,000,000 sheep in 26 counties to 46 cases per 1,000,000 sheep in Makueni County. The highest recorded incidence for cattle (235 cases per 1,000,000 cattle) was much higher than the peaks in other species. The range for camels was narrower, with a maximum of 122 cases per 1,000,000 camels in Tana River. Sheep and goats showed high heterogeneity with generally lower maximum incidence rates compared to cattle and camels.

Over the period, human incidence exhibited a relatively stable trend over time, with occasional fluctuations observed. Conversely, cattle and camel incidence showed sporadic peaks and troughs. Goat incidence exhibited a gradual increase over the study period, indicating a possible emerging health concern within this species. Sheep incidence, on the other hand, remained consistently low throughout the observation period.

The mean incidence rate in humans stood at 10,993 cases per million people, while for animals, it was 1 case per million sheep, 3 cases per million goats, 3 cases per million cattle, and 1 case per million camels, as shown in Table 1.

Overall, 3,630,551 (77.43%) of the reported human cases of brucellosis underwent laboratory diagnosis. Out of all reported animal cases, 24.8% (301/1214) were laboratory-diagnosed, only 0.01% (17/1214) were diagnosed through postmortem, and most, 73.8% (896/1214), were clinically diagnosed, as shown in Table 1 below.

The maximum incidence rate in humans was 14,764 cases per million people. Among animal species, goats had the highest maximum incidence rate of 10 cases per million goats followed by cattle, camels, and sheep with maximum incidence rates of 10, 3, and 2 cases per million population, respectively.

**Figure 2:**
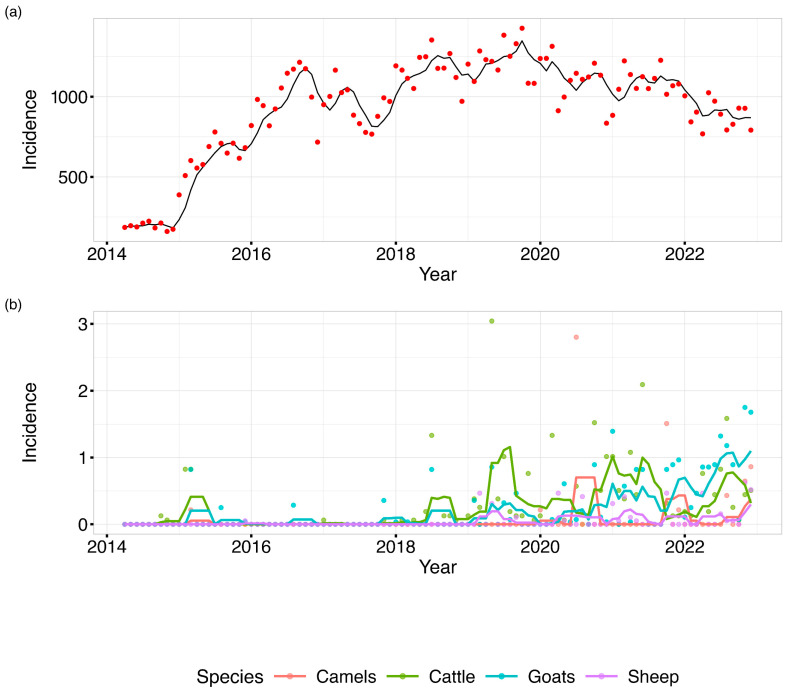
A line graph depicting monthly distribution of (**a**) human and (**b**) animal brucellosis average incidences in Kenya, 2014–2022. (Applied temporal smoothing using a 4-month-period moving average to reduce noise in the monthly incidence time series and facilitate trend identification).

The average brucellosis incidence in humans maintained a gradual increase from around October 2014 and peaked in July 2017. Thereafter, the average incidence showed uneven surges and the greatest peak was recorded in August 2019, as shown in Figure 2. In animals, brucellosis incidence in cattle and goats was seen to follow a fairly similar pattern with the highest incidence being reported in cattle. Cases of brucellosis in camel appeared to have been reported sporadically around 2015, 2019, and 2020. From the trends in the figure below, the trend in animal brucellosis was distinctly different from the trend in human brucellosis incidence.

**Figure 3:**
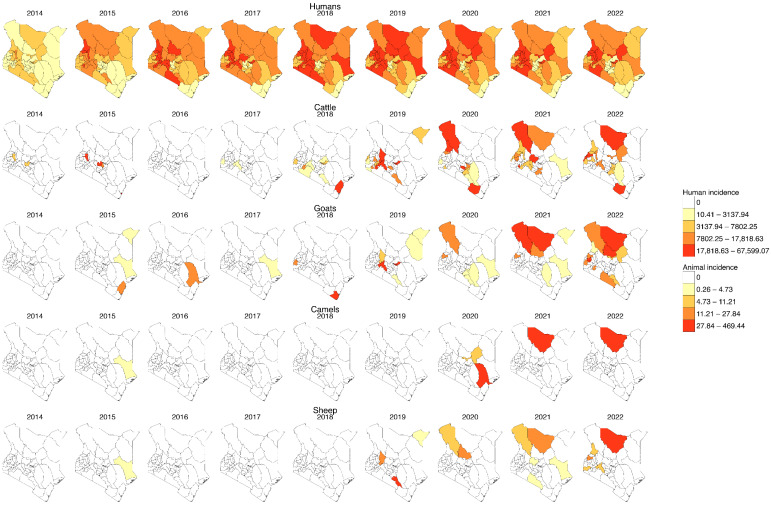
Yearly incidence of human and animal brucellosis at the county level in Kenya, 2014–2022. (For humans, the incidence rate is per 1000 population, while for animal species it is per 1,000,000 population, and the colour is per the percentile. Shapefiles sourced from GADM—https://gadm.org/download_country.html).

Human incidence was higher than animal incidence, with cases distributed across all 47 counties, as shown in Figure 3. The coastal counties consistently reported the lowest incidences of human and animal brucellosis. However, some animal cases were reported in 2015 in goats in Kilifi and in 2018 in cattle. Many counties had no reported cases in animals despite having significant human incidence.

**Figure 4:**
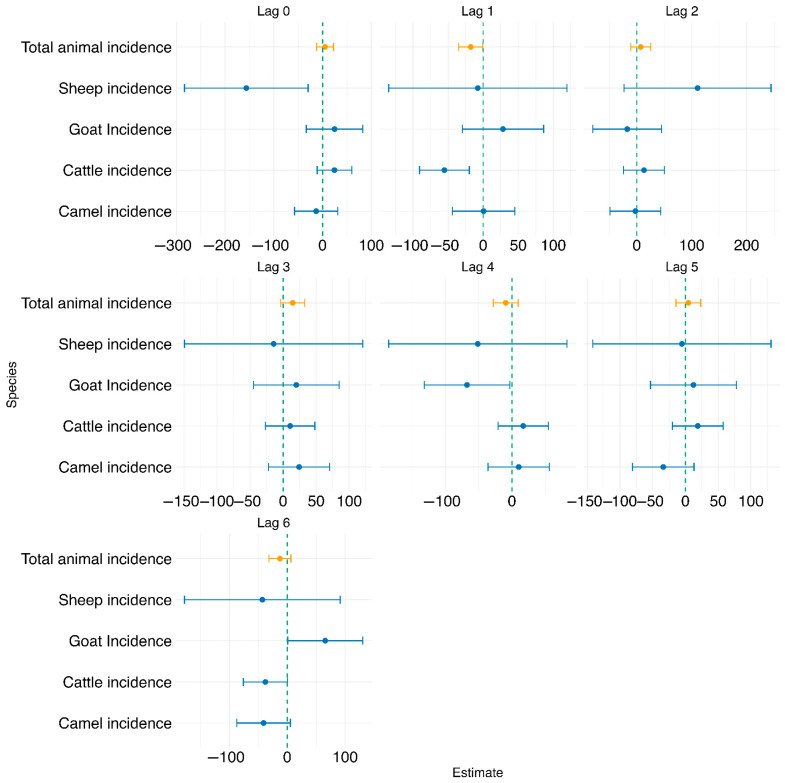
Forest plot showing the test of association between human brucellosis and individual and combined animal species brucellosis at lags 0–6 using the TSLM.

Test of association at national level: As shown in Figure 4 below for individual animal species, at lag 0, sheep incidence was significantly associated with human incidence (estimate = −156.511, *p* = 0.045), while all other species, including total animal incidence, were not significant. At lag 1, cattle incidence showed a significant negative association with human incidence (estimate = −55.345, *p* = 0.012), and total animal incidence was also marginally significant (estimate = −17.817, *p* = 0.090). For subsequent lags (2 to 6), most species-level and total animal incidence variables were not significantly associated with human incidence. However, at lag 4, goat incidence was statistically significant (estimate = −67.529, *p* = 0.087), and at lag 6, goat incidence became positively and significantly associated with human incidence (estimate = 65.600, *p* = 0.099). Across all models, the adjusted R^2^ values remained low (ranging from −0.021 to 0.027), suggesting limited explanatory power. Additionally, model fit metrics such as AIC generally decreased with increasing lag, indicating better model fit at higher lags, although the associations were not consistently significant.

The association between human brucellosis incidence and the log-transformed combined animal brucellosis incidence at lags of 0–6 months was also analyzed, as shown in Figure 4 below. At lag 0, the association was not statistically significant (estimate = 5.061, *p* = 0.631), with a very low adjusted R^2^ of 0.018. At lag 1, total animal incidence showed a marginally significant negative association with human incidence (estimate = −17.817, *p* = 0.090), with a modest increase in model fit (adjusted R^2^ = 0.018, AIC = 1024.73). For lags 2 to 6, the associations remained statistically non-significant (*p* > 0.05), as detailed in Appendix B. Based on model fit statistics and evaluation of the diagnostic plots shown in Appendix A, lag 1 was selected for further analysis, as it demonstrated the strongest statistical association with human incidence (*p* = 0.090), had a lower AIC compared to lag 0, and showed the highest adjusted R^2^ among all lags evaluated. Adjusted R^2^ values for these models were consistently low (ranging from −0.009 to 0.007), indicating limited explanatory power.

Test of association at combined animal species and county level: We further tested the association between combined animal brucellosis incidence and human brucellosis incidence at 0–6 months lag for each individual county. Significant associations between animal and human brucellosis incidence were observed in several counties, with the strongest and most consistent effects occurring at lags 4 to 6 months. Notably, Kajiado, Kilifi, Siaya, and Tharaka Nithi showed strong statistical significance and higher model fit (R^2^).

At lag 0, only weak associations were observed, with marginal significance in Kericho (*p* = 0.052), Nakuru (*p* = 0.062), and Trans Nzoia (*p* = 0.053). Stronger and more statistically significant associations emerged at lag 1, particularly in Nakuru (estimate = −3.47, *p* = 0.009), Narok (estimate = −349, *p* = 0.008), and Tharaka Nithi (estimate = 41.05, *p* = 0.016). At lag 2, significant positive associations were found in Nakuru (estimate = 3.79, *p* = 0.004) and Narok (estimate = 288.8, *p* = 0.031). At lag 3, counties such as Nyeri (estimate = 2.14, *p* = 0.003) and Tharaka Nithi (estimate = −48.7, *p* = 0.004) exhibited strong associations. The trend intensified at lag 4, where several counties demonstrated highly significant associations, including Kajiado (estimate = −810, *p* < 0.001), Siaya (estimate = 20.32, *p* = 0.002), and Tharaka Nithi (estimate = 76.05, *p* < 0.001). The strongest effects were observed at lag 5, particularly in Kajiado (estimate = 1376, *p* < 0.001, R^2^ = 0.36) and Kilifi (estimate = −1.05, *p* = 0.035). At lag 6, Kajiado again showed a significant negative association (estimate = −794, *p* < 0.001), while Kilifi had a strong positive association (estimate = 2.04, *p* < 0.001, R^2^ = 0.165).

**SARIMA prediction analysis:** We used the combined animal incidence data at a lag of 1 month. Two SARIMA models were evaluated for forecasting human brucellosis incidence in 2023: SARIMA(1,1,1)(2,0,0)12 (without a covariate) and SARIMA(2,1,2)(1,0,0)12 (with a covariate). The model without the covariate consistently outperformed the model with the covariate, showing lower RMSE, MAE, and MAPE values. Thus, the simpler SARIMA(1,1,1)(2,0,0)12 model provided a more accurate forecast for human brucellosis in 2023, as shown in Table 2 below.

**Figure 5:**
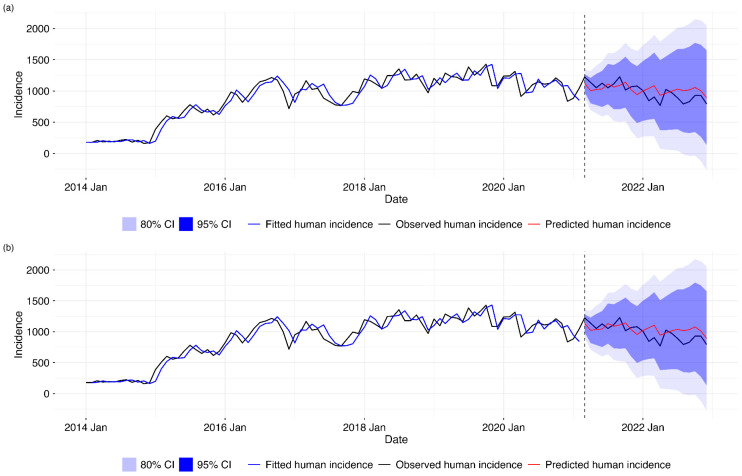
Comparison of SARIMA forecasting models for human disease incidence. (**a**) Univariate SARIMA model using only historical human incidence data. (**b**) SARIMA model with one-month lagged animal incidence exogenous variable. Both panels show observed human incidence (black line) from 2014 to 2021, model-fitted values during the training period (blue line), and out-of-sample forecasts for 2021–2022 (red line) with 80% (dark blue) and 95% (light blue) prediction intervals. The vertical dashed line indicates the start of the forecasting period (March 2021).

A graphical visualization of the model training and forecasts shown in Figure 5 below depicts that our predicted values were within the 80% and 95% prediction interval and that the forecast without the exogenous variable was closer to the actual human incidence. The first plot on the top shows the training and testing forecasting of human incidence based on the first SARIMA model without the exogenous variable. The second plot in the bottom shows the training and testing forecasting of human incidence based on the second SARIMA model with the exogenous variable.

**Figure 6:**
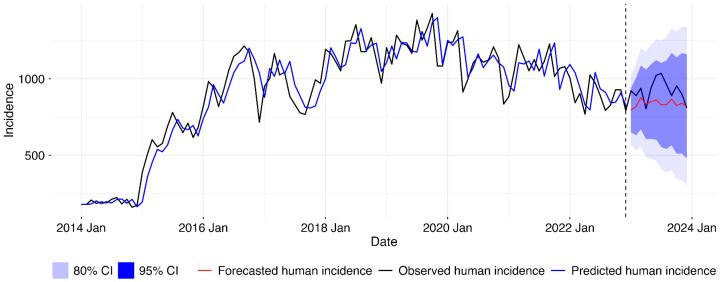
Final SARIMA model for human disease incidence forecasting. Following model comparison, univariate SARIMA model was retrained on full 2014–2022 data and used to generate 2023 forecasts. Lines show observed incidence (black), fitted values (blue), and forecasts (red) with 80% and 95% prediction intervals. Vertical line marks forecast period start.

After establishing that the forecasting model without animal incidence fitted our data better than that with the exogenous variable, we retrained the best model with the complete dataset from 2014 to 2022 and then forecasted for 2023, as shown in the Figure 6 below.

## 4. Discussion

This study uncovers important epidemiological findings that emphasize the need for integrated One Health (OH) surveillance. The number of reported cases of human brucellosis far exceeds the number of animal cases, showing notable regional disparities and variations over time. The spatial distribution suggests that animal cases are being underreported, despite high reported incidences in humans. While we report cases captured by Kenya’s national surveillance system, we acknowledge that the reported human case numbers far exceed global and regional incidence estimates and should be interpreted with caution. Apart from cattle incidence which shows a negative association, there is a lack of a significant association between brucellosis incidences in most animal species and humans, highlighting potential gaps and underreporting in the animal health sector. Additionally, our SARIMA forecasting models demonstrate that although human brucellosis incidence can be reasonably predicted, the inclusion of animal incidence data surprisingly does not significantly improve the predictive power. These findings provide insights into the gaps and potential data quality issues in passive surveillance systems and make a compelling case for policymakers to invest in comprehensive One Health surveillance systems to effectively combat zoonoses.

The human brucellosis case numbers reported by national surveillance systems in this study likely represent substantial overestimations when compared with global and regional incidence estimates [26], primarily due to reliance on clinical diagnoses, and longstanding diagnostic and reporting challenges within Kenya’s surveillance infrastructure. This challenge has been recognized in the national strategy for the prevention and control of brucellosis in humans and animals in Kenya [8].

The clinical picture of brucellosis can be confusing because it overlaps with many other febrile illnesses. For this reason, diagnosis should be supported by laboratory tests [27]. The quality of data from the animal health surveillance system is, therefore, greatly affected by high underreporting and minimal utilization of the laboratory diagnostic services, as evidenced in this study. This compromises the utility of animal brucellosis incidences for predicting human brucellosis.

Brucellosis diagnosis has been identified as one of the major hindrances to disease control [28], since it can be clinically confused with many other conditions in both humans and animals. In our surveillance systems, there were a considerable number of reports that were not confirmed by the laboratory but were diagnosed clinically, especially in the animal health sector. In Kenya, studies have confirmed that lack of reliable diagnostic support, use of highly sensitive but non-specific tests, and routine disease underreporting greatly affect the surveillance systems and potentially cause false alerts [5]. However, there is also a likelihood of overdiagnosis of brucellosis in humans due to the use of rapid tests in most health facilities without confirmatory testing, which leads to possible overestimation of human cases [29]. It is therefore an immediate priority to improve brucellosis surveillance in Kenya and East Africa through the implementation of robust quality control systems and clear guidance on the selection and use of frontline diagnostic tests [8]. Effective integrated surveillance requires harmonized diagnostic testing across human and animal health sectors, as inconsistent test selection undermines data comparability and outbreak detection. Brucellosis serves as a critical model for demonstrating how standardized laboratory confirmation in both sectors coupled with data linkage mechanisms enhances early warning capabilities [30,31].

Brucella organisms localize and multiply in reticuloendothelial tissues like lymph nodes, liver, and bone marrow, which can be sampled postmortem. While postmortem examination is not a primary diagnostic approach, it may enhance bacterial recovery when combined with culture techniques for diagnosing brucellosis [28,32]. In this study, we found that the official human health surveillance system did not capture any cases that may have been identified during the pathological examination postmortem. This could have led to missing important information and underreporting human health events. It is therefore important to consider mortality surveillance, focusing on mortuaries to address this gap.

The observed distinct temporal patterns of brucellosis underscore the complex interplay of factors influencing disease dynamics across each species, highlighting the importance of targeted surveillance and intervention strategies tailored to the specific needs of individual animal populations. We also observed potential correlations in trends between some animal species like goats, and human incidence peaks suggesting possible zoonotic transmission patterns, confirming the fact that brucellosis is an obligate zoonosis. The weak correlations between human and animal case data in most other animal species likely result from incomplete surveillance, diagnostic variability, and complex transmission involving multiple hosts and environmental factors. These challenges, common in pastoralist regions of Kenya, highlight the need for continuous, integrated One Health surveillance to improve our understanding of transmission and support effective control [12,14].

Generally, the spatial distribution and the numbers of cases (reports) captured in the human health surveillance system are comparatively much higher than those captured in the animal health surveillance system. Additionally, the distribution of animal cases does not follow the same spatial pattern as human cases, probably due to poor data quality. This is contrary to previous studies that proved the existence of similar distribution patterns and trends in human and animal brucellosis [33,34,35], and this is probably due to underreporting, misdiagnosis, and other factors weakening the surveillance systems. This may be attributed to the wide distribution and accessibility of numerous health facilities which are the aggregation points for data in the human health sector, unlike the animal health sector where reports are collected at the herd/farm level from villages, laboratories, clinics, crushes, and abattoirs and aggregated at the subcounty level. Additionally, the observed widespread occurrence of human cases across nearly all regions of the country could also be attributed to the fact that people move around far and wide while infected animal products are also traded far and wide, thus spreading infection to many humans.

Cattle and goat incidences follow almost similar distribution patterns and as reported in previous studies, cattle cases were higher than those in sheep and goats [33]. Isiolo County, previously identified as an endemic area for brucellosis by earlier studies [35], did not record any animal cases during the study period. This absence of recorded cases highlights significant weaknesses in the surveillance system [36]. However, the spatial coverage of animal health appeared to improve from around 2018 onwards, an improvement that can be attributed to the national roll out of electronic reporting tools [21].

Most of the cases in the animal health surveillance system were in cattle, since cattle are the most widely distributed animal species in Kenya (KNBS, 2019) [24] and receive the most veterinary attention due to their high economic value. The distribution of brucellosis cases in the country as determined by this study is affected by reporting bias across counties and thus there is a need to ensure that both surveillance systems have a clear process where they report even the absence of priority disease cases or events, called “zero reporting”, to remove uncertainty and distinguish absence of disease [37] from lack of reporting.

Previous studies have established that in developing countries, *B. abortus*, *B. melitensis*, and *B. suis* are the leading causes of animal and human brucellosis [9,27] and that there is a high correlation between human brucellosis and brucella seroprevalence in animals [7,38,39]. However, this study found discrepancies in the association that may be explained by inadequacy and weaknesses in the Kenyan surveillance system and data. In the human health surveillance system, some counties still use manual data collection, contributing to delayed reporting and therefore affecting the data quality, which could contribute to the observed discrepancies. Contrary to our expectations and previous studies, forecast models fitted the data better when brucellosis incidences were forecasted without the animal brucellosis incidences as an exogenous variable, suggesting that the exogenous variable introduced noise in the model, reducing its accuracy and predictive ability, which further underscores the need to address the weaknesses in our surveillance systems. Poor-quality data could arise from challenges affecting the surveillance systems. Some of the key challenges previously identified include the vastness and remoteness of some areas, characterized by poor infrastructure and communications; the need to conduct adequate surveillance with limited financial resources [40]; inadequate numbers of trained personnel [41]; lack of supplies, materials, transport facilities, and diagnostic facilities [42,43,44]; competition among stakeholders driven by financial needs, called “commercial interest” [45]; unharmonized reporting structures; inadequate response to disease outbreaks; lack of incentives and motivations for surveillance; and political interference [43], among others.

Among the practical steps towards addressing the identified challenges in brucellosis surveillance are investing in integrated community-based and One Health surveillance systems that leverage mobile and digital technologies, strengthening workforce capacity through targeted training, and promoting multisectoral coordination. Improving infrastructure, standardizing diagnostic protocols, and implementing zero-reporting mechanisms are essential to ensure data quality and harmonization across human and animal health sectors. Sustained stakeholder engagement further supports resilient and effective surveillance systems capable of early warning and control of zoonotic diseases like brucellosis [46,47].

In line with the principles of One Health and the existence of human–animal and environmental interactions in Kenya’s epidemiological triad, our study agrees with previous research on the importance of addressing priority zoonotic diseases. This includes focusing on disease control in animal reservoirs and strengthening surveillance and management efforts in animals. By doing so, we aim to reduce the incidence of these diseases in human populations through the implementation of a One Health framework [28]. Effective control measures would benefit from a targeted intervention policy and strengthened cooperation between human health and veterinary services [34,48] and would be bolstered by better-quality data.

This study had several limitations. Firstly, the quality of surveillance data may be affected by limited diagnostic capacity, including reliance on clinical diagnosis and inconsistent laboratory testing for brucellosis, which increases the risk of false-positive and false-negative case reporting and thus affects the results. The use of retrospective data extracted from routine surveillance systems could be affected by reporting bias, incomplete records, and potential underreporting, which may diminish the representativeness of findings and influence geographic or temporal patterns. This study also assumes that patients generally seek healthcare services in their respective counties; however, patient movement across counties is possible, which could influence estimates of disease clustering and spatial distribution.

It is important to note that the primary aim of this study was not to validate clinical or laboratory diagnoses, but rather to evaluate the strengths and shortcomings of the official surveillance systems as they currently function in Kenya. Therefore, all data were extracted, cleaned, and analyzed in their original form as reported to the national authorities, providing insights into surveillance system performance under real-world operational conditions. These limitations reinforce the importance of strengthening diagnostic capacities and harmonizing case definitions to enhance the accuracy and effectiveness of integrated zoonotic disease surveillance.

## 5. Conclusions

This study provides the first short-term prediction of human brucellosis in Kenya using national surveillance data. We characterized the spatial distribution, tested the association between human and animal brucellosis, and demonstrated that prediction of a zoonotic disease such as brucellosis is possible. Moreover, we observed that SARIMA is effective in forecasting the human incidence of brucellosis. SARIMA outperforms ETS and ARIMA in forecasting accuracy. Using the established $SARIMA\left(2,1,2\right)\left(1,0,0\right)12$ model, we tested the accuracy of human brucellosis incidence prediction in Kenya from 2022 to 2023. Our study methods and prediction results can contribute to policymakers improving the human and animal diseases surveillance system and can enhance the utility of these surveillance data to strengthen early warning systems.

Animal health surveillance data are important for informing the human health surveillance system and therefore can be useful in forecasting public health events. However, the current surveillance systems, as revealed by this analysis, may not accurately reflect the true disease burden due to underreporting, possible misdiagnosis, and the identified structural and operational issues. These challenges and identified gaps should be addressed to improve the quality of information collected and consequently their potential to inform the early warning systems for zoonoses in Kenya.

These findings reinforce the need for improving the diagnosis and reporting of zoonoses and the potential adoption of the integrated One Health (OH) surveillance approach to enhance synergy across sectors and improve the accuracy of disease reporting and forecasting, ultimately leading to more effective interventions and reduced zoonotic disease burden in Kenya.

## Figures and Tables

**Table 1:** Summary of estimates of brucellosis incidence and methods of brucellosis diagnosis used for cases reported in the national human and animal surveillance systems in Kenya for the period 2014–2022.

Species	Total Cases	Average Annual Cases	Clinically Confirmed	Lab-Confirmed	Postmortem	Mean Incidence Rate	Minimum Incidence Rate	Median Incidence Rate	Maximum Incidence Rate	SD of Incidence Rate
Human	4,688,787	520,976.3	1,058,236 (22.57%)	3,630,551 (77.43%)	0 (0%)	10,992.68	2292.39	11,968.15	14,764.17	3918.56
Cattle	427	47.4	270 (63.23%)	140 (32.79%)	17 (3.98%)	3.01	0	1.97	6.47	2.74
Camel	32	3.6	30 (93.75%)	2 (6.25%)	0 (0%)	0.77	0	0	3.02	1.15
Goat	656	72.9	514 (78.35%)	142 (21.65%)	0 (0%)	2.6	0	1.11	10.32	3.47
Sheep	99	11	82 (82.83%)	17 (17.17%)	0 (0%)	0.57	0	0.05	1.86	0.72

**Table 2:** Error metrics used to select the best fit model.

Model	MAE	RMSE	MAPE
Model with no exogenous variable	108.88	123.44	11.63
Model with exogenous variable	110.16	127.63	11.90

**Figure A1:**
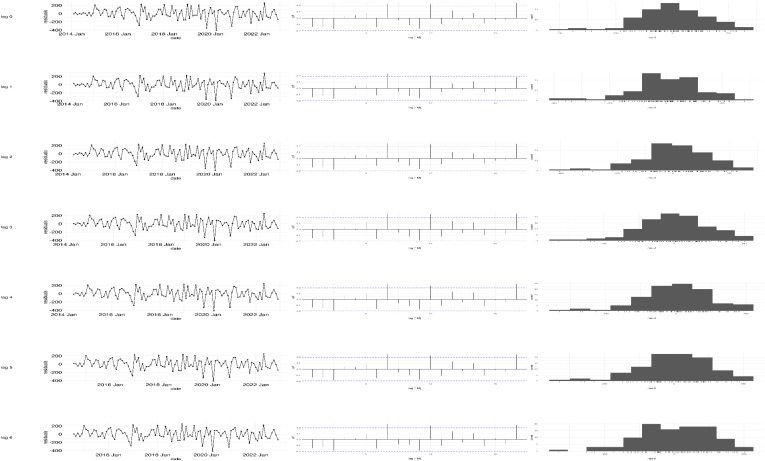
TSLM diagnostics plot with 0–6 months of lag (**left** panel—time series plot; **middle** panel—ACF plot; **right** panel—histogram).

## Data Availability

The data used in this study and the analysis code are available on GitHub at https://github.com/wagathu/brucellosis accessed on 12 November 2025.

## Abbreviations

The following abbreviations are used in this manuscript:

SARIMA	Seasonal Autoregressive Integrated Moving Average
KHIS	Kenya Health Information System
KABS	Kenya Animal Bio-surveillance System
KNBS	Kenya National Bureau of Statistics
TSLM	Time Series Linear Model
ADF	Augmented Dickey–Fuller

## Appendix B

**Table A1:** Tabulation of the error metrics (MAE, RMSE, and MAPE) for the TSLM from lags 1 to 6.

Lag	MAE	RMSE	MAPE
Lag 0	107.340785	121.775861	11.412123
Lag 1	110.164144	127.631526	11.9027991
Lag 2	110.096513	124.752796	11.7661673
Lag 3	106.71078	121.684391	11.3373693
Lag 4	110.259697	122.84345	11.4226258
Lag 5	113.204553	131.292121	12.2466865
Lag 6	99.0380344	118.30954	10.2856198
